# Research on Digital Business Model Innovation Based on Emotion Regulation Lens

**DOI:** 10.3389/fpsyg.2022.842076

**Published:** 2022-03-09

**Authors:** Shan Lu, Haijing Yu

**Affiliations:** School of Business and Management, Jilin University, Changchun, China

**Keywords:** emotion regulation, digital business model innovation, entrepreneurship, environmental dynamism, digital technology

## Abstract

Digital technologies, such as artificial intelligence, brain-computer interfaces technology and big data, enable many firms to innovate their business model. It is clearly an emotional process due to its complex and uncertain nature, and involves individuals’ emotion regulation, yet the current research lacks an effective conversion path from emotion to digital business model innovation (BMI). Drawing on theories and research on emotion regulation and business model innovation, we investigate how emotion regulation of entrepreneurs (i.e., cognitive reappraisal and expressive suppression) influence digital BMI. Data from 126 new ventures show that entrepreneurs’ reappraisal positively affects digital BMI, while entrepreneurs’ suppression exerts opposite effects on digital BMI. Moreover, we find that environmental dynamism moderates this relationship. The findings explain the emotional complexity in digital technology empowerment, which has implications for the development and design of brain computer interface applications and the literature on emotions and business model innovation.

## Introduction

Digital technologies, such as artificial intelligence, brain-computer interfaces technology and big data, are changing entrepreneurial behaviors and outcomes fundamentally, including recognize and regulate emotion using advanced artificial intelligence brain-computer interface to avoid the bias caused by subjective emotion, and creating a new business model (BMI) through the combination of BMI with digital technology, which is labeled as *digital BMI* (e.g., Fichman et al., 2014; Remane et al., 2017). We follow Soluk et al. (2021) in defining it as “a significantly new way of creating and capturing the business value that is embodied in or enabled by digital technologies.” Practice shows that more and more new ventures have achieved rapid growth through digital BMI. Examples of digital business model innovators include Tik Tok, which develops a unique business proposition through building social networking and video-sharing platforms to deliver a new way to create value for consumers (Ma and Hu, 2021). Similarly, some theoretical studies have also revealed the importance of digital BMI to long-term business success (Remane et al., 2017; Sorescu, 2017; Teece, 2018). However, as yet, we have a limited understanding on how emotion influence digital BMI emerge (Soluk et al., 2021). To address this gap, we explore the factors that influence digital BMI from the individual-level emotion lens.

Prior research has indicated the importance of individual entrepreneurs for BMI, focusing on how BMI is triggered by individual-level cognition, including creativity, mindfulness, or analogical reasoning (Snihur and Zott, 2020). However, few studies have explored the emergence of BMI from an emotional lens. This lack of research is especially surprising, since innovation, including BMI, is ambiguous, uncontrollable event (Huy, 2005) laden with uncertainty and potentially affects, positively or negatively, which can trigger emotional ambivalence of entrepreneurs and their stakeholders (Ashforth et al., 2014). Therefore, entrepreneurs need to recognize and regulate their own and stakeholders’ emotions to achieve emotional resonance for innovation adoption. In particular, we propose that entrepreneurs need more emotional regulation in the context of the digital BMI than conventional BMI. The reason is that the constantly changing attribute of digital technology embedded in digital BMI triggers continuous experiment and implementation, which makes the innovation process and results more uncertain (Nambisan et al., 2017). Such higher complexity makes digital BMI trigger more intense emotional experiences of entrepreneurs and stakeholders.

In this regard, we explore how different emotion regulation strategies influence digital BMI. More specially, we focus on two of the most well-established emotion regulation strategies, namely cognitive reappraisal, an antecedent-focused approach, which shape emotion-inner experience by changing the way we see things, and expressive suppression, a response-focused approach relating to inhibiting emotion-external expression (Webb et al., 2012; Gross, 2013). We propose that entrepreneurs’ cognitive reappraisal positively affects digital BMI, while expressive suppression strategies negatively affect digital BMI. We further posit that that such effect of emotion regulation strategies on digital BMI can be shifted by environmental dynamism. We believe that this factor may exert important effects because past research suggests that the effects of emotion regulation is largely context dependent (De Cock et al., 2020). Environmental dynamism refers to the rate of unpredicted change occurring within a given industry (Duncan, 1972; Dess and Beard, 1984). In particularly, such rapid and unpredictable change is more obvious in the digital context (Nambisan, 2017; Lu et al., 2021a; Zhao and Zhou, 2022). Research has shown that entrepreneurs in dynamic environments often experience high levels of stress and anxiety (Hmieleski and Baron, 2008; Shan and Lu, 2020). On the basis of these considerations, we reason that the environmental dynamism, and the emotions that environmental dynamism may evoke, will influence the effectiveness and usefulness of emotion regulation.

We contribute to the present status of research in three main ways. First, we contribute to emotion research in entrepreneurship by investigating the link between emotion regulation and digital BMI. We address the calls for more attention to be paid to the role of emotion regulation in the entrepreneurial process by previous research (e.g., Burch et al., 2013; De Cock et al., 2020; Sirén et al., 2020). While the widespread agreement that the entrepreneurial process is seen as an emotional rollercoaster, entrepreneurs’ emotion regulation has not received much attention. Second, we contribute to research on digital BMI by extending the emotion lens on digital BMI by showing that emotion regulation can be a nuanced explanation of the mechanisms leading to digital BMI. Third, we contribute to the emotion regulation literature by investigating when and how it influence digital BMI in the specific context of entrepreneurship. We find the dynamics of the entrepreneurial environment as boundary conditions for the relationship between emotion regulation and digital BMI. Our theoretical model integrates individual factors and environment factors to explain how certain aspects of emotion regulation become more or less beneficial given different dynamic levels of the entrepreneurial environment.

## Theoretical Background and Hypotheses

### Taking an Emotive View on Digital Business Model Innovation

Business model innovation is defined as “designed, novel, non-trivial changes to the key elements of a firm’s business model and/or the architecture linking these elements” (Foss and Saebi, 2018, p. 201). In recent years, with technological change and global competition, many firms have reshaped the industry pattern and created growth myths through BMI, becoming an essential driving force of innovation-driven development (Johnson, 2010; McGrath, 2010; Afuah, 2014; Hartmann and Vanpoucke, 2017; Narayan et al., 2021). Given its economic importance, BMI has gained increasing attention from academics and practitioners (Foss and Saebi, 2017; Snihur and Zott, 2020; Bhatti et al., 2021; Zhang et al., 2021). In their comprehensive review on BMI, Foss and Saebi (2017) indicated that BMI studies have been growing rapidly since it was first explicitly discussed in Mitchell and Coles (2003). However, with the exception of a few studies, little is known about the possible antecedents of BMI (Foss and Saebi, 2017; Bhatti et al., 2021; Zhang et al., 2021). For example, in their meta-analytic review on BMI, Zhang et al. (2021) suggested that the external factors include market opportunity, situational factors, value network and technology innovation can impact BMI. In addition, individual-level cognition, internal resources and capabilities, as well as organization characteristics as internal factors can foster BMI. In recent studies, there is some new evidence that entrepreneurs influence BMI, including CEO leadership (Colovic, 2021), founder imprinting (Snihur and Zott, 2020), top management mindfulness (Bhatti et al., 2021). Despite the role of entrepreneurs is increasingly studied, there is still a lack of both theoretical and empirical research on BMI from individual entrepreneur’s emotive lens. To address this gap, we draw on the recently developed emotive approach to digital BMI, which suggests that entrepreneurs need to regulate their emotions in the process of developing digital BMI.

Past research has shown that entrepreneurs experience more extreme emotions in the innovation process and that using appropriate emotion regulation strategies to deal with these extreme fluctuating emotions can facilitate innovation adoption and implementation (Huy, 2005; Raffaelli et al., 2019; Shan et al., 2021). More specially, innovation involves mobilizing sufficient resources and challenges members’ basic assumptions about the organization, which define intersubjective reality and provide a way of dealing with ambiguous, uncontrollable events (Schein, 1992; Yu et al., 2020). Organization members are emotionally invested in these non-negotiable assumptions, which challengers their cognitive and emotional stability for core identity, thus, triggers strong defense mechanisms, such as anxiety and defensiveness (Schein, 1992; Huy, 1999). Moreover, emotion theory also predicts a similar emotional response to innovation, which suggests emotions arise from the appraisal of events that are concerning personal goals and important on some valued dimension (Pirola-Merlo et al., 2002; Morris et al., 2012; Davis et al., 2017). If people appraise events as leading to potential benefits, positive emotions are aroused, whereas negative emotions are aroused if they appraise the events as potentially harmful (Huy, 2005; Davis et al., 2017). In this regard, innovation, as a major event of organizational change, is related to entrepreneurs’ goals and organization value creation, which can trigger entrepreneurs’ assessment of whether it’s an opportunity or a threat (Saebi et al., 2017), thus, produce positive or negative emotions. In particular, the digital BMI process experiences more uncertainty, discontinuity and complexity than the traditional innovation process (Soluk et al., 2021). Conventional BMI usually remain in place for several years, while digital BMI involves repeated cycles of experimentation and implementation as the result of the constantly changing nature of digital technologies inherent in digital BMI (Nambisan et al., 2017). Moreover, compared with conventional BMI, digital BMI is also associated with more rapidly changing customer needs and market developments (Sorescu, 2017). Therefore, entrepreneurs are expected to experience greater emotional experience in the digital BMI process than in the traditional innovation process. Thus, we argue that how entrepreneurs regulate their emotions will have an impact on whether their ventures will adapt digital BMI, or conversely, abandon.

### Emotion Regulation and Entrepreneurship

Emotion regulation refers to “attempts to influence which emotions one has, when one has them, and how one experiences or expresses these emotions (Gross, 2015).” According to the time series of emotion generation process, Gross and John (2003) proposed that people could choose five emotion regulation strategies sequentially. These strategies are (1) situation selection, (2) situation modification, (3) attention deployment, (4) cognitive change (cognitive reappraisal), and (5) response modulation (e.g., expressive suppression). The first four of these strategies belong to antecedent-focused emotion regulation strategies, which refers to changing individual’s emotional experience before the emotion is fully evoked. The last one is a response-focused emotion regulation strategy, which refers to altering the individual’s emotional response once it is fully underway. We focus on two different types of strategies—cognitive reappraisal and expressive suppression, as the most commonly used emotion regulation strategies in daily life, which have been most established, intensively studied in psychological research (Gross and John, 2003; Webb et al., 2012; Gross, 2013). More specially, cognitive reappraisal refers to shape emotional experience by altering one’s own perceptions of a situation before emotions are generated. Expressive suppression refers to inhibiting emotion-expressive behavior after emotions are generated (Gross and John, 2003). We expect that these two different types of strategies will have different impacts on digital business model innovation.

Emotion regulation was first proposed in developmental psychology studies in the 1980s (Gross, 1999). In recent years, with the increasing of emotion research in entrepreneurship, some studies began to focus on emotional regulation in entrepreneurial activities. For example, in their case study, Huy and Zott (2019) investigated the effect of emotion regulation on resource mobilization. They found that managers’ emotion regulation of the self (defined as the regulation of one’s own emotion) helps them mobilize human capital resources by creating psychic benefits, whereas their emotion regulation of others (defined as emotion regulation of other stakeholders such as investors and employees) helps mobilize social capital by facilitating legitimacy judgments. In their empirical study. De Cock et al. (2020) investigated how entrepreneurs’ two well-established types of emotion regulation-cognitive reappraisal and expressive suppression influence the likelihood of their venture surviving. They found that cognitive reappraisal lowers their survival odds when ventures are low-performing. In contrast, expressive suppression is generally associated with lower survival chances, except when the venture’s performance is very low, in which case suppression actually significantly increases the survival odds. Sirén et al. (2020) hypothesized and found that in a nascent venture team, an individual with a stronger tendency to reappraise emotions is more likely to emerge as a leader, whereas an individual with stronger tendency to suppress emotions is less likely to emerge as a leader. Moreover, positive team emotions negatively moderate the relationship between reappraisal and leader emergence, whereas a team’s negative emotions magnify the positive relationship between reappraisal and leader emergence.

In sum, as yet, the empirical studies of emotion regulation in an entrepreneurial context are scarce (Huy and Zott, 2019; De Cock et al., 2020). Moreover, the previous scant research on emotion regulation in entrepreneurship has highlighted and empirically demonstrated the importance of this concept for several domains of entrepreneurial outcomes (e.g., resource mobilization, venture survival and leader emergence). However, as yet there is no empirical evidence on the role that emotion regulation play in new venture digital BMI. In the following, we will derive our hypotheses regarding this relationship based on previous evidence from entrepreneurship and the emotion research in psychology.

### Emotion Regulation and Digital Business Model Innovation

First, we expect cognitive reappraisal of entrepreneurs is positively related is digital BMI. As a kind of antecedent-focused emotion regulation strategy, cognitive reappraisal can change an individual’s internal emotional experience by changing how we interpret emotional events (Gross and John, 2003; De Cock et al., 2020; Sirén et al., 2020). For example, an entrepreneur may initially interpret project failures as the end of an entrepreneurial career, and then reappraise it as an opportunity to learn and grow. According to hedonic models of wellbeing, individuals prefer to use cognitive reappraisal strategy to experience more positive emotions and reduce negative emotions (Gross, 2015).

Previous studies have shown that individuals who use cognitive reappraisal are more likely to reframe threats as opportunities in ambiguous contexts (De Cock et al., 2020). The reprogrammable and re-combinable nature of digital technologies inherent in digital BMI enables repeated cycles of experimentation and implementation, making it unclear as to when a particular phase starts and/or ends (Nambisan et al., 2017; Soluk et al., 2021). Such uncertainty induces digital BMI might be perceived as both a threat and an opportunity to the entrepreneur (Saebi et al., 2017). Prior research has shown that the perceived opportunity of managing is related to BMI adaption, whereas perception of threat is related to resisting BMI (Dewald and Bowen, 2010). Thus, we expect that cognitive reappraisal is positively associated with BMI because reappraisers tend to reframe digital BMI as an opportunity rather than a threat. Moreover, many experimental studies on emotion regulation have shown that individuals using cognitive reappraisal strategies experienced more positive emotions and fewer negative emotions (e.g., Gross, 1998; Ray et al., 2010; Lieberman et al., 2011; Szasz et al., 2011; Wolgast et al., 2011; Feinberg et al., 2012). Such positive effect that is often interpreted as a sign that “all is going well” encourages the entrepreneurs to expand their repertoires of useful skills and the range of their social networks, which improves the dynamic capacity of entrepreneurs to respond effectively to environmental change (Baron, 2008; De Cock et al., 2020). Such dynamic capacity has been demonstrated to be crucial for digital BMI (Soluk et al., 2021).

In contrast, we expect expressive suppression of entrepreneurs is negatively related is digital BMI. Expressive suppression, as a kind of response-focused emotion regulation strategy, refers that a person decreasing emotion-expressive behavior but not the internal experience of the emotion while emotionally aroused (Gross, 2014). For example, entrepreneurs may hide their feelings of sadness when faced with a negative event, such as a failed investment. Thus, although expressive suppression maintains the stability of emotions and avoids the spread of emotions among stakeholders, it leads to more negative internal experience (De Cock et al., 2020). Experimental evidence suggested that the person who uses suppression experience less positive emotion and more negative emotion, including painful feelings of inauthenticity as well as depressive symptoms (Gross and John, 2003; Moore et al., 2008; Nezlek and Kuppens, 2008). Such negative emotions are associated with less risk-taking behavior, lower levels of alertness and less engagement in search activities, all of which are important antecedents of innovation (Baron, 2008; Tang et al., 2012; Foo et al., 2015; De Cock et al., 2020). Moreover, prior research has shown that suppression leads to the unreal perception of the entrepreneur by stakeholders, which in turn leads to less social interaction and hinders the establishment of positive relationships with stakeholders (Butler et al., 2003; Gross, 2014; De Cock et al., 2020). However, the development of digital BMI depends on the participation of distributed actors, including external stakeholders (Yoo et al., 2010; Faraj et al., 2011; George et al., 2021). Therefore, we expect that suppression hinders the development of digital BMI by inhibiting the social relationships with stakeholders.

Thus, we hypothesize:

**Hypothesis 1a.** There is a positive relationship between the cognitive reappraisal of a new venture’s entrepreneur and digital BMI.
**Hypothesis 1b.** There is a negative relationship between the expressive suppression of a new venture’s entrepreneur and digital BMI.

### Moderating Effects on Environmental Dynamism

Dynamic environments are characterized by unpredictable and rapid change, which increases uncertainty for entrepreneurs and firms implementing entrepreneurial activities within them (Duncan, 1972; Dess and Beard, 1984; Ensley et al., 2006; Hmieleski and Baron, 2008, Hmieleski and Baron, 2009). Because of such uncertainty, entrepreneurs working in dynamic environments often suffer from heavy information processing burdens. As a result, they may experience high levels of distress and anxiety (Markman et al., 2005; Ensley et al., 2006; Hmieleski and Baron, 2008, Hmieleski and Baron, 2009). Thus, when entrepreneurs develop digital BMI in a highly dynamic environment, they seek approaches to protect themselves against the negative emotions. Emotion regulation are often seen to afford such protection (Gross and John, 2003). Therefore, in a highly dynamic environment, entrepreneurs are encouraged to use emotion regulation strategies to reduce stress and anxiety. Drawing on this line of thinking, we suggest that high environmental dynamism encourages entrepreneurs to use emotion regulation that might help them deal with uncertain times. Thus, we expect that the effect of both emotion regulation strategies on digital BMI is strengthened by the dynamic environment.

Moreover, as the high-level environmental dynamism makes it difficult for organizations to assimilate and anticipate environmental conditions and increase the difficulty of developing digital BMI, firms need to develop multiple solutions to deal with the uncertainty in this process. Firms thus need to encourage their employees to express their ideas and free communication to inspire more ideas and solutions (Ashforth and Humphrey, 1995; Akgün et al., 2008). Previous studies have shown that how entrepreneurs regulate their emotions affects how employees regulation their emotions (De Cock et al., 2020). Entrepreneurs who use cognitive reappraisal create more positive emotions, encouraging communication and expression in ventures. However, new and potentially better opportunities are less likely to be discussed openly within the venture under suppressing emotions (De Cock et al., 2020).

Thus, we hypothesize:

**Hypothesis 2a.** The positive relationship between cognitive reappraisal and digital BMI is strengthened by environmental dynamism.
**Hypothesis 2b.** The negative relationship between expressive suppression and digital BMI is strengthened by environmental dynamism.

## Materials and Methods

### Sample and Data Collection

To test our hypotheses, we used a questionnaire survey from China firms. We chose China as our research context given that digital technology is driving the development of Chinese firms. Moreover, the innovation-driven development, international competition, and digital transformation challenges of key industries in China make this country an ideal context to study digital BMI and the role of environmental dynamism.

We used a sample of Chinese new ventures within 10 years (Milanov and Fernhaber, 2009). To implement the survey, we used the online survey platform of China (i.e., Questionnaire Star). Before sending the survey link to the potential participants, we piloted and pretested the survey through two steps: (1) face-to-face interviews with three entrepreneurial scholars and three entrepreneurs who put forward opinions on possible problems about the questionnaire. (2) sending the web survey to another 25 entrepreneurs to assess the survey structure, wording, and overall length, thus following previous survey-based research (e.g., Obal, 2017). The two pretesting phases led to a final questionnaire.

We collected a total of 200 questionnaires. Further, we excluded those questionnaires that filled out by firms over 10 years and with incomplete information, and finally there were 126 valid questionnaires left, reflecting an effective response rate of 63%. 87 samples (69.05%) are from traditional manufacturing, 13 samples are from IT industry which account for 10.32%. The remaining 26 samples are from other industries, accounting for 20.63%. In terms of the number of employees, 90 samples (71.43%) have less than 200 employees and 36 samples (28.57%) have more than 200 employees.

### Variables

#### Digital Business Model Innovation

We measured digital BMI using nine items derived from Soluk et al. (2021). Participants answered each question on a 7-point Likert-type scale ranging from 1 (strongly disagree) to 7 (strongly agree) (Cronbach’s alpha = 0.90).

#### Emotion Regulation

We measured cognitive reappraisal and expressive suppression using Gross and John’s (2003) 10-items scale. Cognitive reappraisal sub-scale includes six items such as “I control my emotions by changing the way I think about the situation” (Cronbach’s alpha = 0.90). Expressive suppression sub-scale includes four items, such as “I control my emotions by not expressing them” (Cronbach’s alpha = 0.87). Respondents rated their level of agreement with each item using a 7-point Likert-type scale ranging from 1 “strongly disagree” to 7 “strongly agree.”

#### Environmental Dynamism

We measured environmental dynamism using the scale developed by Jansen et al. (2006). The scale is comprised of four items in total. All the items were measured using a 7-point Likert-type scale, with response categories ranging from “strongly disagree” to “strongly agree.”

#### Control Variables

We chose control variables that were also controlled in prior studies on (digital) BMI, including firm’s financial performance (respondents were asked to indicate how successful in terms of ROA their company on a five-point scale: 1 = very unsuccessful; 5 = very successful) (Soluk et al., 2021). We also controlled firm size through the number of employees (1–20, 21–50, 51–200, 201–500, and more than 500) and firm age using the number of years the firm has existed (Guo et al., 2016; Soluk et al., 2021). Finally, we controlled industry by setting three dummy variables (traditional manufacturing, IT, and other industries). These data were collected as demographic items at the end of the administered survey.

### Common Method Bias

This study employed two statistical methods to check the potential of common method bias. First, we took a Harman’s one-factor test and the result showed that the first principal component encompassed only 29.785% of the total variation (less than 40%), thus excluding the existence of common homology bias (Podsakoff et al., 2003). Second, we conducted a confirmatory factor analysis (CFA) to compare a model with all items loading on one latent factor (one-factor model) with our four-factor model). The results shown that our model (χ^2^/*df* = 1.393; comparative fit index [CFI] = 0.944; root mean square error of approximation [RMSEA] = 0.056) indicates superior fit over the one-factor model (χ^2^/*df* = 4.936; CFI = 0.428; RMSEA = 0.177) as indicated by the fit indices (Hu and Bentler, 1999).

### Reliability and Validity

We tested the reliability and validity of the scales (Table 1). The results showed that the Cronbach’s alpha coefficient of each variable was over 0.7, indicating that the scales had good reliability. Furthermore, we tested the composite reliability (CR) and convergent validity of the sample using a CFA. The results show that factor loading for the variables is more than 0.6. In addition, the CR of each variable is higher than 0.8, and the average variances extracted (AVE) for each construct is higher than 0.5 (Fornell and Larcker, 1981). Thus, CR and convergent validity are demonstrated by the CFA results as well. Finally, the correlation coefficients between variables are all less than the square root of AVE, indicating good discriminant validity.

**TABLE 1:** Reliability and validity.

Variables	Items	Factor loading	Cronbach’s alpha	CR	AVE
Cognitive reappraisal (CR)	CR1	0.827	0.896	0.897	0.592
	CR2	0.775			
	CR3	0.757			
	CR4	0.738			
	CR5	0.720			
	CR6	0.795			
Expressive suppression (ES)	ES1	0.804	0.872	0.875	0.636
	ES2	0.834			
	ES3	0.756			
	ES4	0.794			
Digital BMI (DBMI)	DBMI1	0.680	0.902	0.902	0.507
	DBMI2	0.682			
	DBMI3	0.715			
	DBMI4	0.689			
	DBMI5	0.731			
	DBMI6	0.758			
	DBMI7	0.669			
	DBMI8	0.793			
	DBMI9	0.684			
Environmental dynamism (ED)	ED1	0.695	0.868	0.869	0.626
	ED2	0.806			
	ED3	0.809			
	ED4	0.846			

### Descriptive Analyses and Correlations

As shown in Table 2, there is a significant correlation between independent variables and dependent variable. First, cognitive reappraisal is positively associated with digital BMI (*R* = 0.32, *p* < 0.01). Second, expressive suppression is negatively associated with digital BMI (*R* = −0.39, *p* < 0.01). Meanwhile, to eliminate the multi-collinearity between variables, we calculate the variance inflation factor (VIF) of each model. The results show that all of the values are less than 2 and thus multi-collinearity is not a serious problem.

**TABLE 2:** Description statistics and correlation coefficients.

	*M*	*SD*	1	2	3	4	5	6
Firm age	6.300	2.489	1					
Firm size	3.170	0.901	–0.026	1				
Financial performance	2.890	1.045	0.044	–0.006	1			
Cognitive reappraisal	4.370	1.241	–0.084	0.108	0.058	1		
Expressive suppression	3.919	1.173	0.071	0.140	0.182*	0.341**	1	
Environmental dynamism	4.786	1.053	0.017	–0.070	0.184*	0.137	0.149	1
Digital BMI	4.662	1.232	–0.032	–0.080	0.016	0.320**	−0.394**	0.427**

*p < 0.05, **p < 0.01. Dummy variables of the industry are not shown in the table.

### Hypotheses Testing

We model a hierarchical regression to test our hypotheses (in Table 3).

**TABLE 3:** Regression models of emotion regulation and digital BMI.

Digital BMI						
	Model1	Model 2	Model 3	Model 4	Model 5	Model 6
**Control variables**						
Firm age	–0.034	0.049	0.045	0.042	0.027	0.012
Firm size	–0.076	–0.059	–0.016	–0.023	–0.024	–0.039
Industry	–0.059	0.056	0.054	0.073	0.060	0.094
Financial performance	0.021	0.085	0.013	0.008	–0.001	–0.018
**IVs**						
Cognitive reappraisal		0.544***	0.496***	0.520***	0.478***	0.506***
Expressive suppression		−0.584***	−0.627***	−0.614***	−0.637***	−0.620***
**Moderating variable**						
Environmental dynamism			0.450***	0.458***	0.460***	0.478***
**Interaction**						
Cognitive reappraisal × Environmental dynamism				0.123*		0.200**
Expressive suppression × Environmental dynamism					−0.128*	−0.204**
*R* ^2^	0.011	0.405	0.595	0.609	0.610	0.642
Adj-*R*^2^	–0.021	0.375	0.571	0.582	0.583	0.614
*F*	0.344	13.513***	24.758***	22.790***	22.887***	23.108***

*p < 0.05, **p < 0.01, ***p < 0.001.

Hypotheses 1a and 1b predicted that cognitive reappraisal and expressive suppression, respectively, have relationships with digital BMI. As shown in Model 2 of Table 3, there were significant effect of cognitive reappraisal on digital BMI (β = 0.544, *p* < 0.001), thus Hypothesis 1a was supported. Meanwhile, in Model 2, expressive suppression had a significantly negative effect on digital BMI (β = −0.584, *p* < 0.001). Thus, Hypothesis 1b was supported.

**FIGURE 1:**
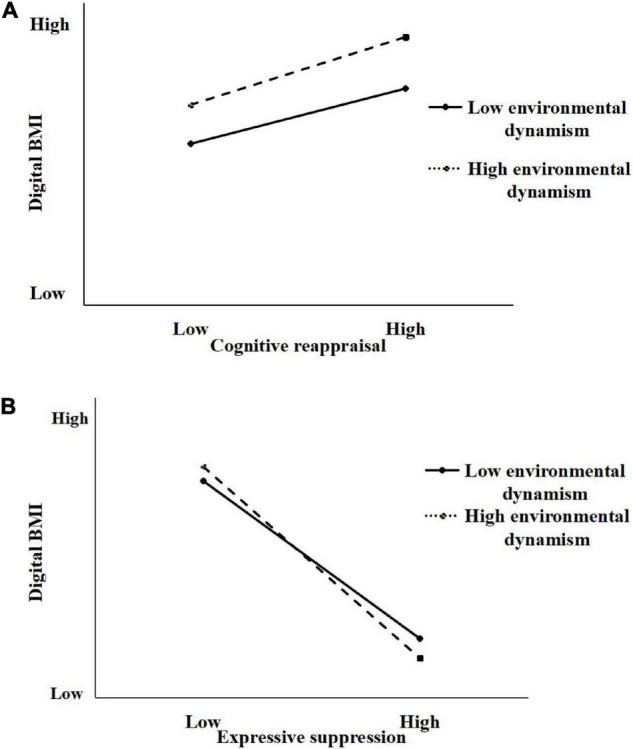
**(A)** Cognitive reappraisal × environmental dynamism interaction (H2a). **(B)** Expressive suppression × environmental dynamism interaction (H2b).

Next, we tested the moderating effect of environmental dynamism on the relationship between emotion regulation and digital BMI (Hypotheses 2a and 2b). As presented in Table 3, we entered the moderator environmental dynamism and the interaction of emotion regulation (cognitive reappraisal and expressive suppression) and environmental dynamism in Model 4 and Model 5. The results in Model 4 shown that the interaction items of cognitive reappraisal and environmental dynamism (β = 0.123, *p* < 0.05) was significant. To facilitate the interpretation of this interaction effect, we visualize the interaction in Figure 1A. As shown in the figure, when environmental dynamism was high, the positive effect of cognitive reappraisal on digital BMI was significant, hypothesis 2a thus was supported. The results in Model 5 shown that the interaction items of expressive suppression and environmental dynamism (β = −0.128, *p* < 0.001) was significant. Meanwhile, the graph of interaction of expressive suppression and environmental dynamism revealed (see Figure 1B) that as environmental dynamism go from low to high, the negative relationship between expressive suppression and digital BMI is amplified, which supported Hypothesis 2b.

## Discussion

### Discussion and Implications for Research

The widespread agreement that entrepreneurial process is seen as an emotional roller coaster. However, studies of emotion regulation in entrepreneurial contexts are scarce. Our study theorized and tested the role of entrepreneurs’ emotion regulation in new ventures’ digital BMI, which offers two novel findings on how and when the emergence of BMI from emotion lens. First, we find that entrepreneurs’ cognitive reappraisal is positively related to digital BMI, whereas entrepreneurs’ expressive suppression has a negative relationship with digital BMI. Second, we predicted and found that the positive impact of cognitive reappraisal on digital BMI is strengthened by environmental dynamism. Also, the negative impact of expressive suppression on digital BMI is strengthened by environmental dynamism.

Our findings contribute to the existing literature in the following ways. First, we investigate the link between emotion regulation and digital BMI in the entrepreneurial context in response to the call of scholars (cf. Burch et al., 2013; De Cock et al., 2020; Sirén et al., 2020) to focus on the role of emotional regulation in entrepreneurship. While the widespread agreement that the entrepreneurial process is seen as an emotional roller coaster, entrepreneurs’ emotion regulation has not received much attention.

Second, we contribute to research on digital BMI by extending the emotion lens on digital BMI by showing that emotion regulation can be a nuanced explanation of the mechanisms leading to digital BMI. Although digital BMI has attracted more and more attention from scholars, there are still few existing studies on the antecedents of business model innovation. This paper explores digital BMI from the perspective of emotion, providing new insights for business model innovation research.

Third, by investigating the moderating role of environmental dynamism in the relationship between emotion regulation and digital BMI, we contribute to the emotion regulation literature by investigating when and how it influence digital BMI in the specific context of entrepreneurship. In particular, we find the dynamics of the entrepreneurial environment as boundary conditions for the relationship between emotion regulation and digital BMI. Our theoretical model integrates individual factors and environment factor to explain how certain aspects of emotion regulation become more or less beneficial given different dynamic levels of entrepreneurial environment, offering a new perspective for further exploring the role of emotion regulation in the entrepreneurial process.

### Implications for Practice

Digital technologies have broken down the structural boundaries of the product and service (Nambisan, 2017; Lu et al., 2021b). In such context, firms that only operate technological innovation or product innovation cannot meet the ever-changing needs of customers, they also need to provide new solutions and create new values for customers through digital BMI (Teece, 2010, 2018). However, many firms are facing the challenge of digital innovation. Through empirical research, we find that the digital BMI of new ventures is influenced by entrepreneurs’ emotional regulation, where cognitive reappraisal emotion promotes digital BMI, while expressive suppression emotion inhibits it. Therefore, entrepreneurs can use cognitive reappraisal emotion consciously to gain the support of stakeholders, thus promoting the adoption and implementation of new business models. In addition to provide useful knowledge for using digital BMI, understanding how entrepreneurs regulate their emotions is also important for employees and team members, which enables them to better understand the decisions of entrepreneurs, thus reducing team conflict and increasing communication efficiency.

### Limitations and Future Research

Several limitations should be taken into consideration. First, we only focus on two types of emotion regulation (i.e., cognitive reappraisal and expressive suppression), other types of emotion regulation (e.g., attention deployment) may also play a role in digital BMI. Further research may build on our study by investigating the relationship between other important emotion and business model innovation. In addition, studies have shown that individuals can use a variety of emotional regulation at the same time (Gross, 2015). Therefore, future research can explore the impact of combination and sequence of different emotion regulation strategies on the entrepreneurial process. Third, our findings have shown that emotion regulation has a critical effect on digital BMI, which reminds us entrepreneurs or firms should pay attention to the emotional aspects in innovation process. Since advanced digital technologies can help entrepreneur to recognize and regulate own and others’ emotion, so it will be an interesting question that how the digital technologies affect the relationship between individual emotion and innovation. Finally, the data in this study are cross-sectional, making it difficult to assert causality. Therefore, we call for longitudinal studies that resolve this problem.

## Conclusion

In conclusion, our study offers valuable insights on the emergence of digital BMI from emotion lens. We show that cognitive reappraisal and expressive suppression-two different emotion regulation—influence digital BMI, and that this relationship is moderated by environmental dynamism. We believe that our findings promise important insights into our understanding of digital BMI and further explores the role of other emotional mechanism in various entrepreneurial activities.

## Data Availability Statement

The raw data supporting the conclusions of this article will be made available by the authors, without undue reservation.

## Ethics Statement

The studies involving human participants were reviewed and approved by the Department of Technology Economy and Management, Jilin University. The patients/participants provided their written informed consent to participate in this study. Written informed consent was obtained from the individual(s) for the publication of any potentially identifiable images or data included in this article.

## Author Contributions

Both authors listed have made a substantial, direct, and intellectual contribution to the work, and approved it for publication.

## Conflict of Interest

The authors declare that the research was conducted in the absence of any commercial or financial relationships that could be construed as a potential conflict of interest.

## Publisher’s Note

All claims expressed in this article are solely those of the authors and do not necessarily represent those of their affiliated organizations, or those of the publisher, the editors and the reviewers. Any product that may be evaluated in this article, or claim that may be made by its manufacturer, is not guaranteed or endorsed by the publisher.
